# Functional significance of nuclear export and mRNA binding of meiotic regulator Spo5 in fission yeast

**DOI:** 10.1186/1471-2180-14-188

**Published:** 2014-07-15

**Authors:** Naoyuki Togashi, Akira Yamashita, Masamitsu Sato, Masayuki Yamamoto

**Affiliations:** 1 Kazusa DNA Research Institute, 2-6-7 Kazusa-kamatari, Kisarazu, Chiba 292-0818, Japan; 2 Department of Biophysics and Biochemistry, Graduate School of Science, University of Tokyo, 7-3-1 Hongo, Tokyo 113–0033, Japan; 3 Department of Life Science and Medical Bioscience, Graduate School of Advanced Science and Engineering, Waseda University, 2-2 Wakamatsucho, Shinjuku, Tokyo 162-8480, Japan; 4 National Institute for Basic Biology, Nishigonaka 38, Myodaiji, Okazaki, Aichi 444-8585, Japan

**Keywords:** Fission yeast, Meiosis, RNA export, RNA-binding protein, ATF/CREB family

## Abstract

**Background:**

Meiotic cells undergo two rounds of nuclear division and generate gametes. Previous studies have indicated that a number of transcription factors modulate the transcriptome in successive waves during meiosis and spore formation in fission yeast. However, the mechanisms underlying the post-transcriptional regulation in meiosis are not fully understood. The fission yeast *spo5*^*+*^ gene encodes a meiosis-specific RNA-binding protein, which is required for the progression of meiosis II and spore formation. However, the target RNA molecules of Spo5 are yet to be identified. Characterization of meiosis-specific RNA-binding proteins will provide insight into how post-transcriptional regulation influence gene expression during sexual differentiation.

**Results:**

To assess the functional significance of RNA-recognition motifs (RRMs) of Spo5, we constructed a series of new *spo5* truncated mutants and previously reported *spo5* missense mutants. In addition, we isolated novel *spo5* missense mutants. The phenotypic characteristics of these mutants indicated that the RRMs are essential for both the localization and function of the protein. Interestingly, Spo5 is exported from the nucleus to the cytoplasm via the Rae1-dependent mRNA export pathway, but is unlikely to be involved in global mRNA export. Furthermore, cytoplasmic localization of Spo5 is important for its function, which suggests the involvement of Spo5 in post-transcriptional regulation. We identified *pcr1*^*+*^ mRNA as one of the critical targets of Spo5. The *pcr1*^*+*^ gene encodes an activating transcription factor/cAMP response element binding (ATF/CREB) transcription factor family. Among the four family members, namely Pcr1, Atf1, Atf21, and Atf31, only the mRNA encoding Pcr1 binds to Spo5.

**Conclusions:**

Spo5 is exported from the nucleus with mRNAs via the Rae1-dependent pathway. RRMs are necessary for this process and also for the function of Spo5 after the nuclear export. Spo5 appears to influence the activity of *pcr1*^*+*^ mRNA, and the mechanism of how Spo5 stimulates the mRNA to promote the progression of meiosis II and spore formation remains an intriguing question for future research.

## Background

Meiosis is a specialized cell division process, which includes premeiotic DNA synthesis, DNA recombination followed by two rounds of cell division, and gametogenesis [1-4]. It has been shown in the fission yeast *Schizosaccharomyces pombe*[5-8] and the budding yeast *Saccharomyces cerevisiae*[1,9-12] that a number of transcription factors dramatically modulate the transcriptome to facilitate meiosis, thereby playing critical roles in meiotic progression and sporulation (gametogenesis). In addition to transcriptional regulation, post-transcriptional regulation plays a fundamental role in the progression of meiosis and gametogenesis in higher eukaryotes. For example, cessation of transcription followed by complex translational activation and repression of stored maternal mRNAs occurs in *Xenopus* oocytes during meiotic progression (oocyte maturation) [13]. In fission yeast, a specialized regulation of meiosis, called selective elimination of meiosis-specific mRNAs, facilitates the post-transcriptional degradation of meiotic mRNAs during the mitotic cell cycle [14]. We were interested in elucidating any additional post-transcriptional regulation that might contribute to the dynamic changes observed in gene expression during meiosis in the fission yeast.

The *spo5* mutant was isolated as a sporulation-deficient mutant in the original genetic screen of defective mutants in meiotic progression and/or sporulation, performed nearly half a century ago [15]. Previous studies have indicated that the *spo5*^+^ gene encodes a meiosis-specific RNA-binding protein, carrying two RNA-recognition motifs (RRMs) in the C-terminal part (aas 192–567), and regulates the progression of meiosis II and spore formation [16-20]. Although it seems apparent that Spo5 plays an essential role to coordinate meiosis and sporulation, controlling a number of targets, the RNA molecules that bind to Spo5 have not yet been identified, except for our recent finding that *cdc13*^*+*^ mRNA, encoding cyclin B, can do so [21]. Because Spo5 is likely to be involved in post-transcriptional events during meiosis, such as pre-mRNA processing, mRNA export, translation, and mRNA degradation, we analyzed this RNA-binding protein to evaluate its functions.

In this report, we demonstrate that the RRMs on Spo5 are essential for its cytoplasmic localization, where it exerts its function. Spo5 appears to be exported from the nucleus to the cytoplasm through the Rae1-dependent mRNA export pathway, but is unlikely to be involved in general mRNA export. We also show that one of the critical target RNA molecules for Spo5 is *pcr1*^*+*^ mRNA. Pcr1 belongs to the ATF/CREB family of transcription factors, which consists of four members in fission yeast: Pcr1, Atf1, Atf21, and Atf31. The functional relationship between Spo5 and Pcr1 is also analyzed.

## Results and discussion

### RNA recognition motifs are essential for the localization and function of Spo5

**Figure 1:**
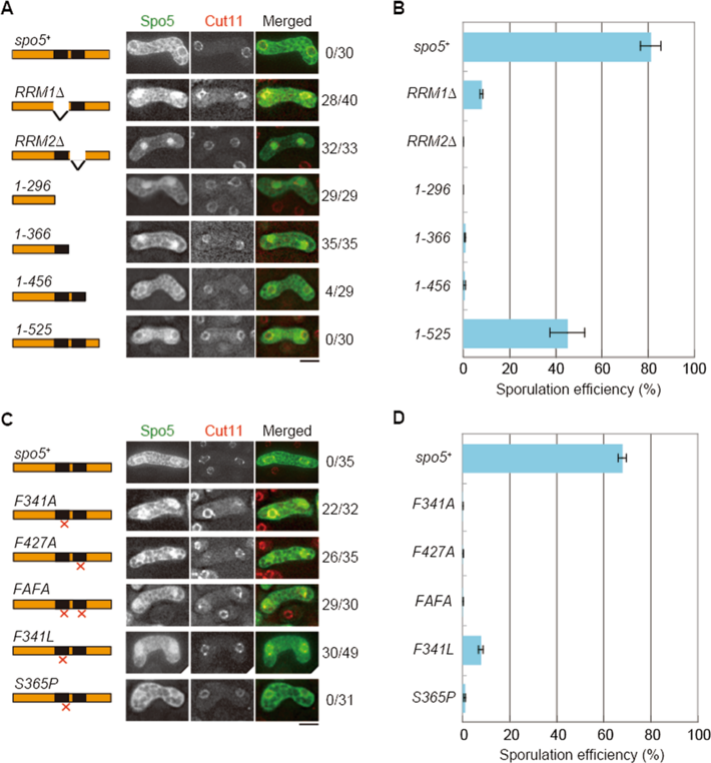
**RNA recognition motifs are essential for the localization and function of Spo5. (A)** Localization analyses using truncated mutants of Spo5. Localization of wild-type and mutant proteins of Spo5-GFP (green) during the period between meiosis I and meiosis II was detected using the nuclear envelope marker Cut11-4mRFP (red). Numbers on the right indicate the frequency of cells displaying nuclear GFP signals. Schematic images depict the domains of the mutant proteins. Black boxes depict two RNA-recognition motifs, RRM1 and RRM2, respectively. Scale bar, 5 μm. **(B)** Sporulation efficiency of specific mutants used in **(A)**, measured at 30°C (n > 500). Error bars indicate standard deviation. **(C)** Localization analyses using point mutants of Spo5. Localization of Spo5-GFP harboring specific point mutations was observed as in **(A)**. Positions of mutation sites (×) are shown in schematic images. Scale bar, 5 μm. **(D)** Sporulation efficiency of the strains used in **(C)**, measured at 30°C (n > 500). Error bars indicate standard deviation.

First, we evaluated the mechanism underlying the subcellular localization of Spo5. A previous study suggested that the C-terminal half of Spo5 is required for its cytoplasmic localization [19]. As this region contains the RRMs, we examined whether these might be responsible for the localization of Spo5. We constructed a series of truncation mutants that lacked the RRM region (Figure 1A). Each mutant carried the mutated *spo5* gene in place of the wild-type gene on the chromosome, and expressed the mutant protein from the authentic *spo5* promoter. All truncated mutant proteins that lacked at least one RRM showed abnormal nuclear accumulation, as indicated by the fluorescent signals of fused green fluorescent protein (GFP) (Figure 1A). We confirmed that mutant proteins were produced in a comparable amount to wild-type Spo5, by examining the shortest mutant Spo5(1–296) and the RRM2-deletion mutant, although the protein level might be slightly lower in the latter case (Additional 1: Figure S1). The RRM truncation mutants were defective in sporulation (Figure 1B), suggesting that the existence of intact RRMs is correlated with the cytoplasmic localization and proper function of Spo5. While we could not exclude the possibility that some of the effect of the mutations on sporulation might be due to lower levels of the mutant proteins, the effect on sporulation was apparently more dramatic than the effect on protein levels, supporting the importance of the RRMs/cytoplasmic localization in Spo5 function.There was one exception, namely Spo5(1–456)-GFP, which has two RRMs but lacks the C-terminal region. Although it localized mostly to the cytoplasm, it was not functional, whereas Spo5(1–525)-GFP, which has two RRMs and an additional C-terminal portion, was functional (Figure 1A,B). This indicates that, in addition to intact RRMs, the C-terminal region adjacent to the second RRM is essential for the Spo5 function.

To clarify whether RNA-binding is required for Spo5 localization and function, we introduced missense mutations to the conserved motifs in RRMs. We constructed mutant strains that were similar to the F341A and F427A mutations previously analyzed by Kasama and colleagues [19]. All of the mutant proteins, namely Spo5(F341A)–GFP, Spo5(F427A)–GFP, and Spo5(F341A, F427A)–GFP, accumulated in the nucleus, as did the RRM-truncated proteins (Figure 1C). These mutants were also deficient in sporulation (Figure 1D). Similar, or slightly lower production of Spo5(F341A, F427A) protein compared to the wild-type was confirmed (Additional file 1: Figure S1). The nuclear localization of these mutated Spo5-GFP proteins in our study was somewhat different from that observed in the previous study, which reported that Spo5(F341A, F427A)-GFP shows a punctate distribution in both the cytoplasm and nucleus [19]. Our precise analysis suggested that Spo5(F341A, F427A) protein might exhibit punctate cytoplasmic distribution during early meiotic stages such as the horsetail-movement and one-nucleus stages (Additional file 2: Figure S2). The apparent difference in localization may also reflect changes in the strain construction: In the previous study, the *spo5* mutant allele was integrated into the chromosomal *leu1* locus, whereas the mutant allele was integrated in the authentic *spo5* locus in our case. However, the actual reasons remain unclear.

We also performed a genetic screen for novel missense mutants of *spo5* that were defective in meiosis and sporulation, and isolated more than ten different mutants including *spo5(F341L)* and *spo5(S365P)*, both of which contained a mutation in the RRM domain. When assayed at 30°C, Spo5(F341L)–GFP accumulated in the nucleus as did Spo5(F341A)–GFP, but Spo5(S365P)–GFP did not, showing a unique phenotype (Figure 1C). Because aromatic residues in the RRM, such as F341, have been shown to interact directly with RNA [22], we speculated that the RNA-binding of Spo5 might be important for its cytoplasmic localization.

### Spo5 localizes to the cytoplasm via the mRNA export pathway

**Figure 2:**
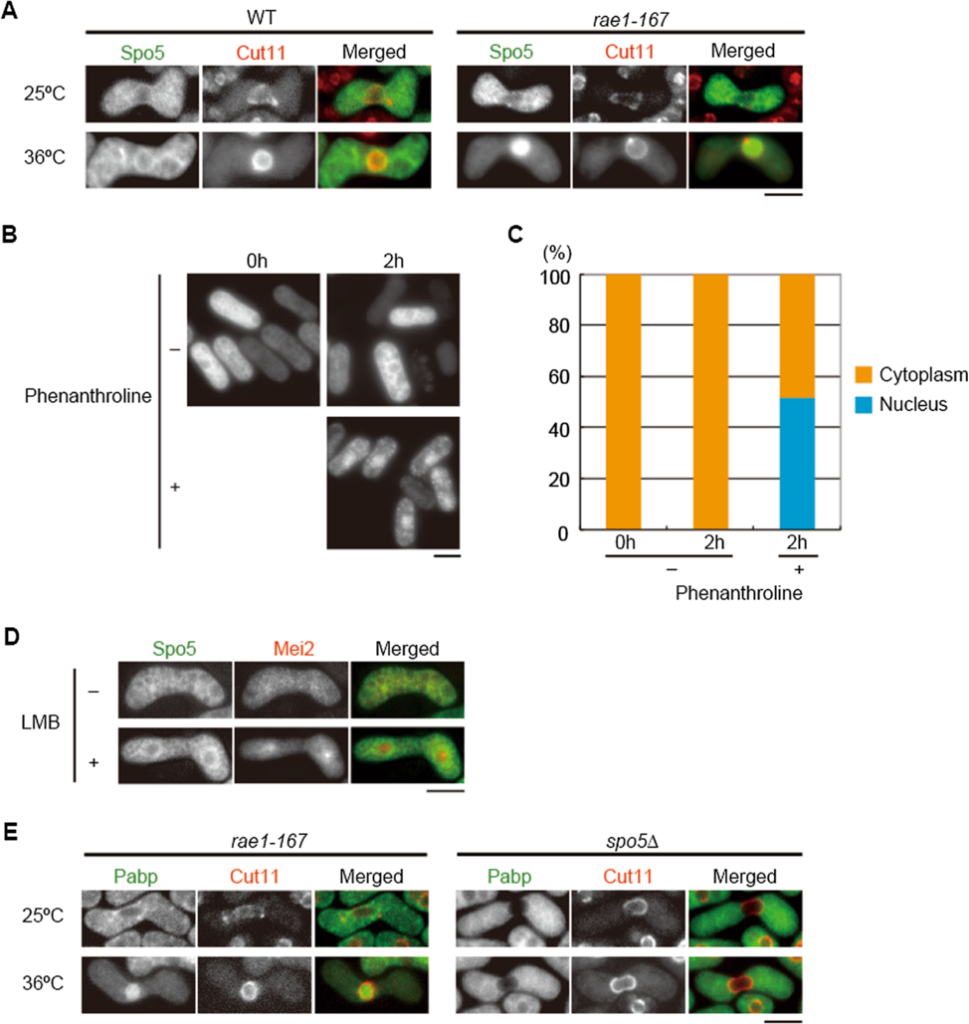
**Spo5 localization to the cytoplasm facilitated by the mRNA export pathway does not involve global mRNA export. (A)** Spo5–GFP accumulated in the nucleus in temperature-sensitive *rae1-167* cells. Cells were incubated at 25°C for 6 h and then transferred to 36°C for 3 h. Cut11-4mRFP was used as a nuclear envelope marker. The Spo5–GFP signal was evident in cells undergoing meiotic prophase I through meiosis II. Scale bar, 5 μm. **(B)** To block RNA-polymerase II-dependent transcription of mRNAs, we used 1, 10-phenanthroline [49]. Wild-type diploid cells were transferred to MM-N to induce meiosis, and after 3.5 hours (Time 0), 1,10-phenanthroline was added to half of the culture at a final concentration of 500 ng/μL. Microscopic observation of Spo5-GFP was carried out after 2 hours. Scale bar, 5 μm. **(C)** Quantitative analysis of Spo5-GFP localization in cells treated with 1,10-phenanthroline. The number of cells examined is as follows: 0 h, n = 152; 2 h(-), n = 239; and 2 h(+), n = 207. **(D)** Cells expressing Spo5–GFP (-LMB) were treated with 100 ng/mL LMB and observed after 1 h (+LMB). Mei2-mCherry served as a positive control since it accumulates in the nucleus upon LMB addition. Scale bar, 5 μm. **(E)** Pabp–GFP accumulated in the nucleus in *rae1-167* cells, whereas it did not do so in *spo5*∆ cells. Scale bar, 5 μm.

RRMs appear to be essential for the translocation of Spo5 from the nucleus to the cytoplasm. Therefore, we investigated whether Spo5 might be exported to the cytoplasm via the mRNA export machinery using a mutant of the mRNA export factor, Rae1. The Rae1-dependent pathway is conserved from the budding yeast [23] to humans [24]. The fission yeast *rae1-167* mutant exhibited defective mRNA export at restrictive temperatures [25]. We induced meiosis in wild-type (WT) and *rae1-167* cells at 25°C, the latter of which produced Spo5 protein in a comparable or slightly lower amount (Additional file 1: Figure S1), and transferred the cells to 36°C to inactivate Rae1-167. In WT cells, Spo5–GFP was mainly localized to the cytoplasm both at 25°C and 36°C (Figure 2A). By contrast, Spo5–GFP accumulated in the nucleus in *rae1-167* cells at the restrictive temperature (Figure 2A). This suggests that Spo5 is likely to be exported to the cytoplasm via the mRNA export machinery. Consistent with this idea, Spo5 accumulated in the nucleus when mRNA synthesis was inhibited by the addition of 1,10-phenanthroline (Figure 2B,C). We also noticed that sporulation was markedly inefficient in *rae1-167* cells (*spo5-GFP rae1*^*+*^, 83% vs. *spo5-GFP rae1-167*, 19%), indicating that Rae1 plays an important role in meiotic progression and sporulation.

Next, we investigated the possible involvement of the exportin-mediated mRNA export pathway [26] in the localization of Spo5. Ran GTPase is known as the major organizer of importin/exportin-mediated nucleocytoplasmic transport [27]. When the Ran–exportin nuclear export pathway was blocked with leptomycin B (LMB), a potent inhibitor of exportin/Crm1 [28], Spo5–GFP remained in the cytoplasm (Figure 2D), demonstrating that exportin/Crm1 is dispensable for the export of Spo5.

To examine whether Spo5 itself is a component of the mRNA transport machinery, we monitored the localization of the poly (A)-binding protein (Pabp), which has been shown to accumulate in the nucleus in the *rae1-167* mutant [29]. Pabp–GFP was found to localize in the cytoplasm in *spo5*∆ cells (Figure 2E), suggesting that Spo5 is not an mRNA export factor.

Taken together, we conclude that Spo5 is exported to the cytoplasm via the mRNA export machinery, but is unlikely to be involved in global mRNA export. It was recently shown that *Drosophila* RAE1 plays an essential role in male meiosis and spermatogenesis [30]. Fission yeast Rae1 may also promote meiosis by transporting mRNPs from the nucleus to the cytoplasm. Some RNA-binding proteins are also known to be exported to the cytoplasm through binding to mRNAs [31-33]. The mRNA-dependent nuclear export of RNA-binding proteins may emerge as a more universal phenomenon among eukaryotes than previously anticipated.

### Nuclear export is important for the function of Spo5

The findings of this study indicated that mutations in RRMs caused abnormal nuclear accumulation and loss of function of Spo5. This suggests that the mRNA-dependent nuclear export of Spo5 is required for its function, although it is also possible that the loss of RNA-binding in Spo5 resulted in its abnormal localization as a secondary effect. Hence, we employed two approaches to investigate whether the nuclear export of Spo5 is a fundamental requirement for its function.

**Figure 3:**
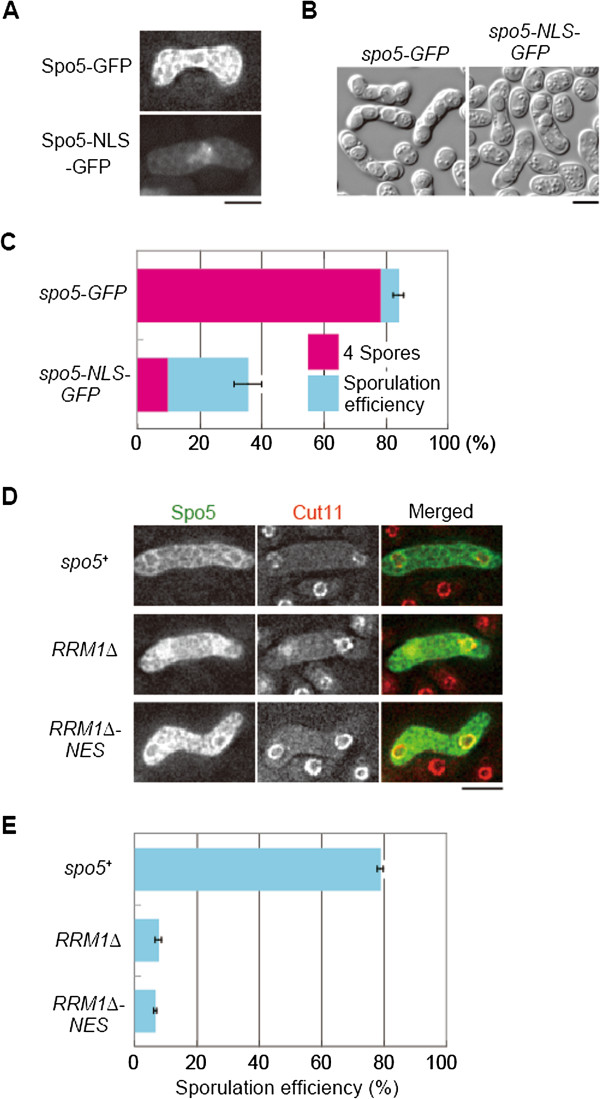
**Nuclear export is important for the function of Spo5. (A)** Spo5 fused with an NLS sequence derived from SV40 large T antigen and GFP (Spo5–NLS–GFP) accumulated in the nucleus during meiosis. Scale bar, 5 μm. **(B)** Addition of the NLS caused deficient sporulation. Differential interference contrast (DIC) images are shown. Scale bar, 5 μm. **(C)** Quantitative representation of the reduction in sporulation efficiency (light blue) and the frequency of four-spore asci (magenta) (*n* > 500). Error bars indicate standard deviation. **(D)** Nuclear accumulation of Spo5(RRM1∆ )–GFP was suppressed by the fusion of the NES sequence. Scale bar, 5 μm. **(E)** Addition of NES did not suppress the sporulation defects of Spo5(RRM1∆ ) (*n* > 500). Error bars indicate standard deviation.

First, we used an external nuclear localization signal (NLS) to alter the localization of Spo5 artificially from the cytoplasm to the nucleus. Although the addition of NLS appeared to make the protein less stable (Additional file 1: Figure S1), cells expressing Spo5–NLS–GFP accumulated the fusion proteins detectably in the nucleus, indicating that the NLS sequence was functional (Figure 3A). The sporulation efficiency of these cells was significantly reduced, and they produced abnormally shaped spores or asci with less than four spores (Figure 3B,C). While the destabilization of Spo5–NLS was likely to contribute largely to the sporulation defect, enforced nuclear migration of Spo5 also appeared to abolish its function to promote meiosis and sporulation, suggesting that the nuclear export of Spo5 is important for its optimal function. The viability of the spores generated in *spo5-NLS-GFP* cells was comparable to that of the control (*spo5-GFP*, 50% vs. *spo5-NLS-GFP* 40%), implying that the loss of Spo5 function did not affect the germination potential.

Second, we investigated whether artificial nuclear export of Spo5 could suppress sporulation defects in some *spo5* mutants. Removal of RRM1 caused nuclear accumulation of Spo5 and sporulation defects, although a small portion of *spo5 RRM1*∆ cells could sporulate (Figure 1A,B). The fusion of a nuclear export signal (NES) to Spo5RRM1∆ (Spo5RRM1∆ –NES–GFP) enhanced its localization to the cytoplasm (Figure 3D). However, the sporulation efficiency was much lower than that observed for Spo5–GFP carrying intact RRM1, and was only comparable to that observed for Spo5RRM1∆ -GFP (Figure 3E). Hence, we conclude that the RNA-binding activity of Spo5 is necessary not only for nuclear export of the Spo5–mRNA complex via the mRNA export machinery, but also for the function of Spo5 in the cytoplasm.

### *pcr1*^*+*^ mRNA is one of the critical targets of Spo5

The *spo5(S365P)* mutant isolated in this study was unique in that it did not accumulate Spo5(S365P) protein efficiently in the nucleus in *rae1-167* mutant cells at the restrictive temperature (Additional file 3: Figure S3A), although it was produced in a comparable or slightly lower amount to the wild-type (Additional file 1: Figure S1), implying that this mutant protein might be defective in nuclear import. In addition, the *spo5(S365P)* mutant was less leaky compared to the *spo5(F341L)* mutant, which sporulated almost normally at 25°C, as indicated by iodine staining [15] and microscopic measurement (Additional file 3: Figure S3B, C). Therefore, we screened for multi-copy suppressors of *spo5(S365P)*, expecting to identify novel candidates. We introduced the cDNA library to the *spo5(S365P)* mutant and picked up iodine-positive colonies. In this screening we obtained clones encoding transcription factors. Among them was the *pcr1*^*+*^ gene, which encodes an ATF/CREB family transcription factor. The *pcr1*^*+*^ gene was not a novel candidate, because we have previously reported the isolation of this gene as a multicopy suppressor of the *spo5* mutant [34]. We and others have characterized Pcr1, which is now known to form a heterodimer with Atf1 and binds to the *ade6-M26* meiotic recombination hotspot [35,36].

**Figure 4:**
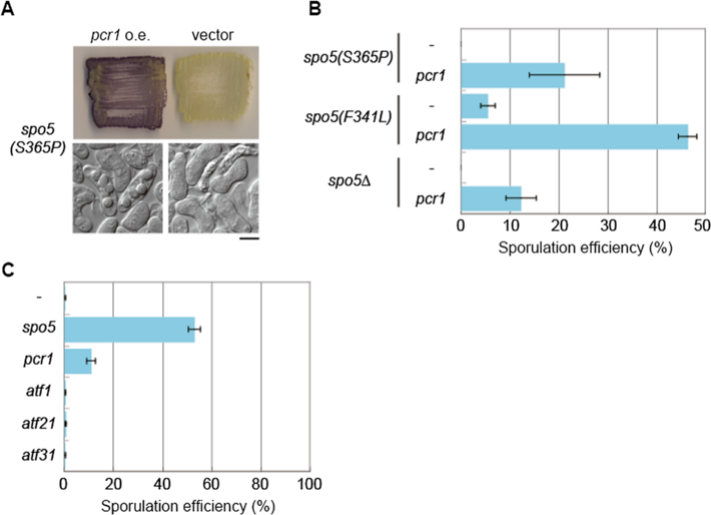
***pcr1***^***+ ***^**mRNA is one of the critical targets of Spo5. (A)** The sporulation of the *spo5(S365P)* strain was detected by dark brown staining with iodine vapor when *pcr1*^+^ was overexpressed. Stained patches and DIC images of the cells are shown. Scale bar, 5 μm. **(B)** Sporulation defects of other *spo5* mutants were also suppressed by the overexpression of *pcr1*^+^. Meiosis and sporulation were induced in cells harboring pPEP1 (vector) or pPEP3-*pcr1*^+^, and the sporulation efficiency was calculated (*n* > 500). Error bars indicate standard deviation. **(C)** Meiosis was induced in *spo5*∆ cells harboring plasmids containing *spo5*^+^, *pcr1*^+^, *atf1*^+^, *atf21*^+^, and *atf31*^+^, or the empty vector on SSA at 30°C for 3 days, and sporulation efficiency was calculated (*n* > 500). Error bars indicate standard deviation.

The relationship between Pcr1 and Spo5, however, has not been delineated. Transcription of *pcr1*^*+*^ was not lowered in the *spo5* null mutant, *spo5*∆ (Additional file 4: Figure S4A). The levels of *pcr1*^*+*^ transcripts gradually increase toward late meiosis [5] (after 4 h in Additional file 4: Figure S4A), suggesting that Pcr1 may also function in a meiotic process other than meiotic recombination. Overexpression of *pcr1*^*+*^ could suppress the sporulation deficiency of *spo5(S365P)* and other *spo5* mutants including *spo5*∆ (Figure 4A,B). These data suggest that Pcr1 may act downstream of Spo5, and that meiotic progression can be promoted substantially without Spo5 if Pcr1 is expressed at sufficiently high levels.

*S. pombe* has four ATF/CREB family proteins, namely Pcr1, Atf1, Atf21, and Atf31 [37]. Although we examined all of them, only Pcr1 could suppress sporulation defects of *spo5*∆ cells when overexpressed (Figure 4C). Curiously, overexpression of Atf1, a functional partner that forms a heterodimer with Pcr1 [35], failed to suppress *spo5* mutants. This suggested that Spo5 might be specifically related to *pcr1*^*+*^ gene expression, and furthermore, that Pcr1 might promote transcription of some meiotic genes that are regulated by Spo5. It was also evident that Spo5 could regulate factors other than Pcr1 that are important for meiotic progression and sporulation, because Pcr1 could not suppress sporulation defects of *spo5*∆ as efficiently as Spo5 itself (Additional file 4: Figure S4B), although *pcr1*^*+*^ overexpression increased the amount of *pcr1*^*+*^ mRNA in *spo5*∆ cells to a higher level than *spo5*^*+*^ overexpression (Additional file 4: Figure S4C). Indeed, we have recently demonstrated that Spo5 is necessary to maintain proper expression of cyclin Cdc13 [21].

**Figure 5:**
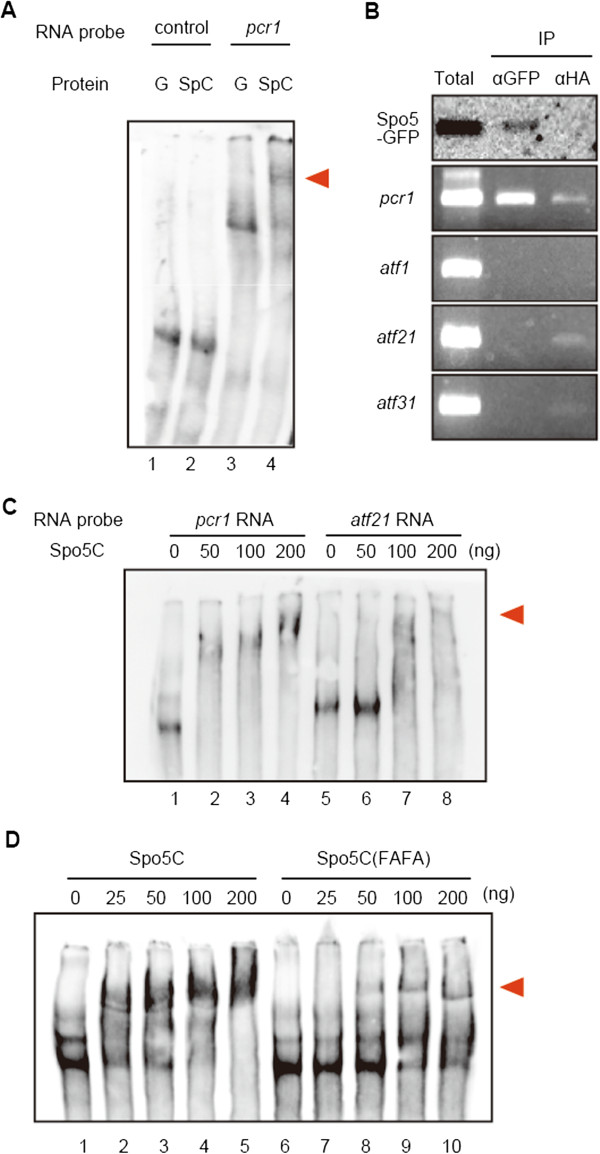
**Binding of Spo5 protein to *pcr1***^**+ **^**mRNA. (A)** Results of an EMSA assay indicating formation of the Spo5–*pcr1*^+^ RNA complex. Recombinant GST and GST–Spo5C (the C-terminal part of Spo5, aas 192–567) proteins were incubated with *pcr1*^+^ RNA including the coding region and both 5′- and 3′-UTRs (lanes 3 and 4) or control *GFP* RNA (lanes 1 and 2). ‘G’ indicates GST (50 ng), and ‘SpC’ indicates GST-Spo5C (20 ng). The red arrowhead indicates shifted RNA. **(B)** Spo5–GFP and *pcr1*^+^ mRNA form complexes *in vivo*. A cell extract was prepared from a diploid Spo5–GFP strain undergoing meiosis and treated with anti-GFP and the control anti-HA. Reverse transcription-polymerase chain reaction (RT-PCR) assay using the pull-downs was performed to detect *pcr1*^+^ and other ATF/CREB factors, *atf1*^*+*^, *atf21*^*+*^, and *atf31*^*+*^ mRNA. **(C)***pcr1*^+^ RNA complexes with Spo5C more efficiently than *atf21*^+^ RNA does. *atf21*^+^ RNA carried the coding region and both 5′- and 3′-UTRs, similarly to *pcr1*^+^ RNA. The red arrowhead indicates shifted RNA. **(D)** Spo5C binds to *pcr1*^+^ RNA more efficiently than the mutant form Spo5C(FAFA), suggesting the involvement of the two phenylalanine residues in RNA binding. The red arrowhead indicates shifted RNA.

To test the possibility that Spo5 may bind to *pcr1*^*+*^ mRNA to modulate its expression/function during meiosis, we performed an electrophoresis mobility shift assay (EMSA) using recombinant Spo5 protein and *pcr1*^*+*^ RNA transcribed *in vitro*. As shown in Figure 5A, the C-terminal part of Spo5, including the two RRMs (Spo5C, aas 192–567), fused to glutathione *S*-transferase (GST) (GST-Spo5C), associated with the *pcr1*^*+*^ RNA (red arrowhead), but not with the control *GFP* RNA. To confirm direct interaction of Spo5 and *pcr1*^*+*^ RNA *in vivo*, we performed an RNA-immunoprecipitation assay using the *spo5-GFP* diploid strain. Immunoprecipitation by anti-GFP antibody indicated that *pcr1*^*+*^ mRNA formed a complex with Spo5-GFP *in vivo*, but that the mRNAs of other ATF/CREB factors did not (Figure 5B). To further confirm the specificity of binding, we compared binding of Spo5C to *pcr1*^*+*^ RNA and *atf21*^*+*^ RNA transcribed similarly *in vitro*. While Spo5C bound to *atf21*^*+*^ RNA to some extent (Figure 5C, lanes 7 and 8), probably because of its weak non-specific affinity for RNA, which we noticed previously [21], it was clear that Spo5C could bind to *pcr1*^*+*^ RNA more strongly than *atf21*^*+*^ RNA, as 50 ng of the protein was enough to shift *pcr1*^*+*^ RNA (Figure 5C, lane 2). We also confirmed that Spo5C carrying the F341A and F427A mutations, designated Spo5C(FAFA), lost the binding ability to *pcr1*^*+*^ RNA (Figure 5D, compare lanes 2 vs. 7, and lanes 3 vs. 8), demonstrating the importance of the RRMs for the binding. These observations indicate that *pcr1*^*+*^ mRNA is one of the critical targets of Spo5. We have shown that *cdc13*^*+*^ mRNA also binds to Spo5 [21]. A previous report suggested that a long 3′ UTR of *cdc13*^*+*^, *cdc25*^*+*^ and *ste9*^*+*^ mRNA might determine its stability [38]. The *pcr1*^*+*^ mRNA also carries a relatively long 3′ untranslated region (UTR), but our observation does not support the idea that Spo5 stabilizes *pcr1*^*+*^ mRNA (Additional file 4: Figure S4A). Thus, it is an interesting hypothesis to be confirmed that Spo5 may control certain activity of *pcr1*^*+*^ mRNA through binding to its long 3′ UTR.

## Conclusions

The findings of this study indicated that Spo5 is exported to the cytoplasm via the mRNA export machinery, but not via the Ran–exportin/Crm1 system. The binding of Spo5 to mRNAs through its two RRMs appears to enable its nuclear export, and this RNA-binding activity is essential not only for the nuclear export, but also for its function in the cytoplasm. Spo5 is unlikely to be involved in general mRNA export, suggesting that it may instead be involved in the post-transcriptional regulation occurring in the cytoplasm, such as control of mRNA stability and/or translational initiation. Furthermore, we identified a novel binding target of Spo5, *pcr1*^+^ mRNA. Overexpression of the *pcr1*^+^ gene suppressed the sporulation deficiency of *spo5* mutants, demonstrating that *pcr1*^+^ mRNA is a critical target of Spo5. However, Pcr1 suppressed the defect in *spo5∆ * cells less efficiently than Spo5, suggesting that Spo5 may also regulate factors important for meiotic progression and sporulation other than Pcr1. Suppression of *spo5* by overexpression of *cdc13*^+^ is also partial [21]. Future studies are required to identify other important targets of Spo5 and the possible role that Spo5 plays in the post-transcriptional regulation of *pcr1*^+^ mRNA and other mRNAs.

## Methods

### Yeast strains and genetic manipulations

The *S. pombe* strains used in this study are listed in Table 1. Conventional methods were used to construct gene-disrupted strains, fluorescent protein-tagged strains [39,40], and a multiple fluorescent protein-tagged strain [41]. To enforce the localization of Spo5 to the nucleus and cytoplasm, the nuclear localization signal (NLS; PKKKRKV) of SV40 large T antigen and the leucine-rich nuclear export signal (NES; ILPPLERLTL) of HIV-1 Rev were used, respectively [42-44]. Standard methods were employed to grow yeast strains [45]. To induce mating, meiosis, and sporulation of homothallic (*h*^90^) strains, sporulation agar (SPA) or synthetic sporulation medium (SSA) was used. Haploid cells grown in liquid yeast extract containing 3% glucose (YE) supplemented with adenine (YEA) were harvested, spotted onto SPA plates, and then incubated at 30°C (Figures 1, 2D and 3, and Additional file 2: Figure S2) and 25°C (Additional file 3: Figure S3B, C). Figure 4A-C illustrates the cells that were streaked on SSA plates and incubated at 30°C. For experiments with temperature-sensitive mutants, cells were spotted onto SPA and incubated at 25°C, and then transferred to 36°C (Figure 2A,E, Additional file 3: Figure S3A). The sporulation efficiency was calculated by counting more than 500 cells under the microscope, and each experiment was repeated three times. We also used *h*^*+*^*/h*^*-*^ diploid cells (Figures 2B,C and 5B and Additional file 4: Figure S4).

**Table 1:** Strains used in this study

**No.**	**Genotype**	**Figures**
NT4	*h*^*90*^*spo5(S365P)-GFP-kan ade6-M216 leu1 ura4*	Figure 1D, Figure 4AB and Additional file 3: Figure S3BC
NT92	*h*^*90*^*spo5(F341L)-GFP-kan ade6-M216 leu1 ura4*	Figure 1D, Figure 4B and Additional file 3: Figure S3BC
NT180	*h*^*90*^*spo5-GFP-kan mei2-mCherry-hph ade6-M216 leu1*	Figure 2D
NT184	*h*^*90*^*spo5-GFP-kan ade6-M216 leu1 ura4*	Figure 1BD, Figure 3A-CE and Additional file 3: Figure S3BC
NT196	*h*^*90*^*spo5(S365P)-GFP-kan cut11-4mRFP-hph CFP-atb2-nat ade6-M216 leu1 ura4*	Figure 1C
NT205	*h*^*90*^*spo5(1–296)-GFP-kan ade6-M216 leu1 ura4*	Figure 1B and Additional file 1: Figure S1
NT207	*h*^*90*^*spo5(1–366)-GFP-kan ade6-M216 leu1 ura4*	Figure 1B
NT209	*h*^*90*^*spo5(1–456)-GFP-kan ade6-M216 leu1 ura4*	Figure 1B
NT403	*h*^*90*^*spo5-GFP-kan cut11-4mRFP-hph CFP-atb2-nat ade6-M216 leu1 ura4*	Figure 1AC, Figure 2A and Figure 3D,
		Additional file 1: Figure S1, Additional file 2: Figure S2 and Additional file 3: Figure S3A
NT648	*h*^*90*^*spo5(1–525)-GFP-kan ade6-M216 leu1 ura4*	Figure 1B
NT716	*h*^*90*^*spo5(1–525)-GFP-kan cut11-4mRFP-hph CFP-atb2-nat ade6-M216 leu1 ura4*	Figure 1A
NT745	*h*^*90*^*spo5(F341L)-GFP-kan cut11-4mRFP-hph CFP-atb2-nat ade6-M216 leu1 ura4*	Figure 1C
NT798	*h*^*90*^*spo5(1–296)-GFP-kan cut11-4mRFP-hph CFP-atb2-nat ade6-M216 leu1 ura4*	Figure 1A
NT799	*h*^*90*^*spo5(1–366)-GFP-kan cut11-4mRFP-hph CFP-atb2-nat ade6-M216 leu1 ura4*	Figure 1A
NT814	*h*^*90*^*spo5(1–456)-GFP-kan cut11-4mRFP-hph CFP-atb2-nat ade6-M216 leu1 ura4*	Figure 1A
NT973	*h*^*90*^*spo5(RRM1∆ , ∆ 297-366)-GFP-kan ade6-M216 leu1 ura4*	Figure 1B and Figure 3E
NT974	*h*^*90*^*spo5(RRM2∆ , ∆ 385-456)-GFP-kan ade6-M216 leu1 ura4*	Figure 1B and Additional file 1: Figure S1
NT1001	*h*^*90*^*spo5(RRM1∆ , ∆ 297-366)-GFP-kan cut11-4mRFP-hph CFP-atb2-nat ade6-M216 leu1 ura4*	Figure 1A and Figure 3D
NT1002	*h*^*90*^*spo5(RRM2∆ , ∆ 385-456)-GFP-kan cut11-4mRFP-hph CFP-atb2-nat ade6-M216 leu1 ura4*	Figure 1A
NT1168	*h*^*+*^/ *h*^*-*^*spo5-GFP-kan/ spo5-GFP-kan ade6-M216/ade6-M210 leu1/leu1 ura4/ura4*	Figure 2BC and Figure 5B
NT1208	*h*^*90*^*spo5(S365P)-GFP-kan rae1-167 ade6-M216 leu1 ura4*	Additional file 1: Figure S1
NT1495	*h*^*90*^*spo5::ura4*^*+*^*::spo5(F427A)-GFP-kan ade6-M216 leu1 ura4*	Figure 1D
NT1568	*h*^*90*^*spo5::ura4*^*+*^*ade6-M216 leu1 ura4*	Figure 4BC and Additional file 3: Figure S3BC
NT1622	*h*^*90*^*spo5::ura4*^*+*^*::spo5(F427A)-GFP-kan cut11-4mRFP-hph CFP-atb2-nat ade6-M216 leu1 ura4*	Figure 1C
NT1632	*h*^*90*^*spo5::ura4*^*+*^*::spo5(F341A)-GFP-kan ade6-M216 leu1 ura4*	Figure 1D
NT1634	*h*^*90*^*spo5::ura4*^*+*^*::spo5(F341A, F427A)-GFP-kan ade6-M216 leu1 ura4*	Figure 1D and Additional file 1: Figure S1
NT1641	*h*^*90*^*spo5::ura4*^*+*^*::spo5(F341A)-GFP-kan cut11-4mRFP-hph CFP-atb2-nat ade6-M216 leu1 ura4*	Figure 1C
NT1648	*h*^*90*^*spo5::ura4*^*+*^*::spo5(F341A, F427A)-GFP-kan cut11-4mRFP-hph CFP-atb2-nat ade6-M216 leu1 ura4*	Figure 1C and Additional file 2: Figure S2
NT1677	*h*^*90*^*spo5-GFP-kan rae1-167 ade6-M216 leu1 ura4*	Additional file 1: Figure S1
NT1743	*h*^*90*^*spo5 + NLS-GFP-kan ade6-M216 leu1 ura4*	Figure 3A-C and Additional file 1: Figure S1
NT1769	*h*^*90*^*spo5-GFP-kan cut11-4mRFP-hph rae1-167 ade6-M216 leu1*	Figure 2A and Additional file 3: Figure S3A
NT1793	*h*^*90*^*pabp-GFP-kan spo5::ura4*^*+*^*cut11-4mRFP-hph ade6-M216 leu1 ura4*	Figure 2E
NT1898	*h*^*90*^*spo5(S365P)-GFP-kan rae1-167 cut11-4mRFP-hph ade6-M216 leu1 ura4*	Additional file 3: Figure S3A
NT1905	*h*^*90*^*pabp-GFP-kan rae1-167 cut11-4mRFP-hph ade6-M216 leu1 ura4*	Figure 2E
NT1922	*h*^*90*^*spo5(RRM1∆ ) + NES-GFP ade6-M216 leu1 ura4*	Figure 3E
NT1943	*h*^*90*^*spo5(RRM1∆ ) + NES-GFP cut11-4mRFP-hph ade6-M216 leu1 ura4*	Figure 3D
NT2130	*h*^*+*^/ *h*^*-*^*ade6-M216/ade6-M210 leu1/leu1 ura4/ura4*	Additional file 4: Figure S4A-C
NT2132	*h*^*+*^/ *h*^*-*^*spo5::ura4*^*+*^*/spo5::ura4*^*+*^*ade6-M216/ade6-M210 leu1/leu1 ura4/ura4*	Additional file 4: Figure S4A-C

The original *rae1-167* strain was provided by Ravi Dhar [25].

### Mutagenesis

To isolate novel *spo5* missense mutants, the parental *spo5–GFP–kan* strain, in which the GFP–T*adh*-*kan* fragment was inserted at the chromosomal *spo5*^+^ locus, was constructed. Genomic DNA was isolated from this strain, and a DNA fragment containing the entire coding region of *spo5*^+^ with *GFP*–T*adh1*–*kan* genes flanked by 500-bp up- and down-stream sequences, was amplified. The amplified fragment was then subjected to error-prone PCR amplification in order to introduce random mutations to the product, using Ex Taq DNA polymerase (Takara Bio; Japan) with 40 rounds of thermal cycling. The mutagenized fragment was introduced to the homothallic (*h*^90^) wild-type strain JY878, and colonies that conferred G418 resistance were selected. Colonies were then replica-plated to SSA plates, and those that were deficient in sporulation were chosen using iodine staining. To exclude nonsense mutants, GFP fluorescence was monitored and GFP-positive colonies were selected. Standard sequencing methods (using a 3130 Genetic Analyzer; Applied Biosystems) were used to determine the mutation sites (F341L and S365P). The PrimeSTAR mutagenesis kit (Takara Bio) was used to introduce the F341A, F427A, and F341A mutations into the *spo5*^+^ gene cloned using the vector pCR2.1-TOPO (Life Technologies; CA, USA). The mutated fragments were then introduced into the *spo5::ura4*^+^ strain, in which the *spo5* coding region was replaced with the *ura4*^+^ cassette. Colonies with correct inserts were selected on YEA plates containing 1 mg/mL 5-fluoro-orotic acid (5-FOA; Wako Pure Chemicals; Japan).

### Plasmids

The plasmids containing the *pcr1*^+^ gene (pREP3–*pcr1*^+^) and the *spo5*^+^ gene (pREP3–s*po5*^+^) were isolated from a cDNA library. The pREP1 plasmids carrying the *atf1*^+^, *atf21*^+^, and *atf31*^+^ genes were a gift from Takatomi Yamada [37], and we subsequently replaced their *nmt1* promoters with the *nmt81* promoter.

### Microscopy

An Axioplan2 fluorescence microscope (Zeiss; Germany) equipped with a CoolSNAP HQ2 CCD camera (Photometrics; AZ, USA) and SlideBook software (Leeds Precision; MN, USA) were used to acquire the images presented in Figures 2, 3A,B and 4A, Additional file 2: Figure S2 and Additional file 3: Figure S3A. Single-sectioned images along the Z-axis were captured and deconvolved. Single-cell imaging was performed using the DeltaVision-SoftWoRx system (GE Healthcare; UK) with a CoolSNAP HQ2 CCD camera, as described previously [41]. Briefly, the cells selected for observation were mounted on a glass-bottomed dish (Matsunami Glass; Japan) precoated with lectin and filled with MM–N liquid medium. Serial-sectioned images were acquired along the Z-axis and stacked using the ‘quick projection’ algorithm in SoftWoRx.

To block the Crm1/exportin-dependent nuclear export machinery, 100 ng/mL leptomycin B (LMB) [28] was added to a culture of Spo5-GFP Mei2-mCherry cells. After 60 min incubation at 30°C, cells were subjected to fluorescence microscopy. Mei2-mCherry, an LMB-sensitive meiotic protein, served as a positive control [46].

The *rae1-167* strain was a gift from Ravi Dhar [25]. We employed this mutant to arrest mRNA export during meiosis. Cells were incubated on SPA at 25°C for 6 h, and then shifted to a restrictive temperature at 36°C for 3 h prior to microscopic observation.

### Electrophoretic mobility shift assay (EMSA) of RNA

As reported previously [47], digoxigenin (DIG)-labeled RNA was prepared from PCR products using the DIG RNA Labeling kit (SP6/T7) (Roche). The RNA-binding reaction was performed using 2 ng of DIG-labeled RNA (control; derived from the GFP sequence, *pcr1* and *atf21*) and 20–200 ng of either recombinant GST, GST-Spo5C (aas 192–567), or GST-Spo5C(FAFA), in 4 μL of a modified KNET buffer consisting of 20 mM KCl, 80 mM NaCl, 2 mM ethylene glycol bis-(2-aminoethylether) tetraacetic acid (EGTA), 50 mM Tris–HCl (pH7.5), 0.05% NP-40, 1 mM MgCl_2_, 2 mM dithiothreitol, 10% glycerol, and RNase Inhibitor (Roche). RNA with the GFP-coding sequence was used as a negative control. Samples were preincubated at room temperature with 10 μg of carrier *Escherichia coli* tRNA for 25 min. Labeled RNA was then added and incubated for another 25 min. Samples were analyzed using polyacrylamide gel electrophoresis and electroblotted to a GeneScreen Plus membrane (NEN) using 0.5× Tris-borate-EDTA (TBE) buffer. Signals were detected using a DIG Luminescent Detection Kit (Roche).

### RNA-immunoprecipitation

Diploid cells expressing Spo5-GFP were cultured in MM + N at 30°C for 15 h, shifted to MM-N (0 h, 30°C) and sampled for RNA extraction after 6 h. Detailed conditions for RNA-IP were described previously [48]. Immunoprecipitated RNA was isolated by phenol-chloroform extraction. For immunoprecipitation, anti-GFP (Roche; GFP monoclonal antibody) and anti-HA (Abcam; 16B12, HA monoclonal antibody) were used. Reverse transcription was performed using the TaKaRa RNA PCR kit (AMV, Ver 3.0; Takara bio).

### Other methods

Western blotting, reverse transcription and quantitative PCR were performed according to the same protocols as we described previously [21]. Following oligonucleotides were used in RT-qPCR: pcr1(forward) CCGAATTCTGGAGCGCAAT, pcr1(reverse) CACTCTTTCTTTTTCTGGCGAAA, act1(forward) TGAGGAGCACCCTTGCTTGT, and act1(reverse) TCTTCTCACGGTTGGATTTGG. Inhibition of mRNA transcription by 1,10-phenanthroline was carried out as described precisely by Galipon et al. [49].

### Availability of supporting data

The data sets supporting the results of this article are included within the article and its additional files.

## Competing interests

The authors declare that they have no competing interests.

## Authors’ contributions

NT performed the experiments and analyzed the results. AY and MS designed the outline of this study and analyzed the results. MY supervised the work. NT, AY, MS, and MY wrote the manuscript. All authors read and approved the final manuscript.

## Supplementary Material

Additional file 1: Figure S1 Expression level of mutant Spo5 proteins under meiotic conditions. Cell extracts were prepared from representative *spo5* mutant strains incubated on SPA at 25 ºC for 8 hours. They were separated by SDS-PAGE and subjected to western blotting. Each Spo5-GFP protein, which was expressed from the authentic *spo5* promoter, was detected with an anti-GFP antibody. α-tubulin was also detected as a loading control. The wild-type strain shown here for comparison carried an additional gene encoding CFP-tagged Atb2, which reacted with both anti-GFP and anti-α-tubulin (lane 1). Click here for file

Additional file 2: Figure S2 Localization of Spo5(FAFA) protein during the progression of meiosis. Localization of WT Spo5 (left) and Spo5(FAFA) (right) was examined at four stages of meiosis, namely horsetail-movement, one-nucleus, two-nuclei, and four-nuclei stages. GFP-tagged Spo5 (green) and the nuclear envelope marker Cut11-4mRFP (red) were detected. Scale bar, 5 μm. Click here for file

Additional file 3: Figure S3 Characterization of a novel *spo5* missense mutant *spo5(S365P)*. (A) Spo5(S365P)–GFP did not accumulate in the nucleus of the temperature-sensitive *rae1-167* cells. Cells were incubated at 25°C for 6 h and then shifted to 36°C for 3 h. Cut11-4mRFP was used as a nuclear envelope marker. (B) At 25°C, WT and *spo5(F341L)* cells were stained dark brown with iodine vapor and sporulated efficiently, while *spo5(S365P)* cells were stained light brown and sporulated weakly. The *spo5∆ * cells showed a white colony and did not sporulate. At 30°C, the two mutants showed nearly white colonies and did not sporulate efficiently, as indicated quantitatively in Figure 1D. (C) Sporulation efficiency of WT and the two *spo5* mutant strains at 25°C (n > 500). Error bars indicate standard deviation. Click here for file

Additional file 4: Figure S4 Expression of *pcr1*^+^ mRNA is not lowered in *spo5*∆ cells. (A) Meiosis was induced in *spo5*^+^ (WT) and *spo5*∆ diploid cells, as in Figure 5B, and total RNA was isolated from them every 2 hours. The relative amount of *pcr1*^+^ mRNA in *spo5*^+^ and *spo5*∆ cells was determined by RT-qPCR. The quantity of mRNA was normalized to the expression level of *act1* mRNA. Error bars show standard deviation. Two independent samples were analyzed for each strain. n = 3, for each sample. (B) Sporulation efficiency was measured for *spo5*∆ cells harboring either the vector, the *pcr1*^*+*^ clone, or the *spo5*^*+*^ clone. (n > 500) (C) The relative amount of *pcr1*^+^ mRNA was determined by RT-qPCR in *spo5*∆ cells harboring either the vector, the *pcr1*^*+*^ clone, or the *spo5*^*+*^ clone. Total RNA was isolated every 4 hours after the induction of meiosis, and analyzed as in (A). Error bars show standard deviation. Click here for file
