# Supplementary figures and images for: Functional significance of nuclear export and mRNA binding of meiotic regulator Spo5 in fission yeast
Source: BMC Microbiol. 2014 Jul 15;14:188. doi: 10.1186/1471-2180-14-188 (PMC4109790; doi:10.1186/1471-2180-14-188)

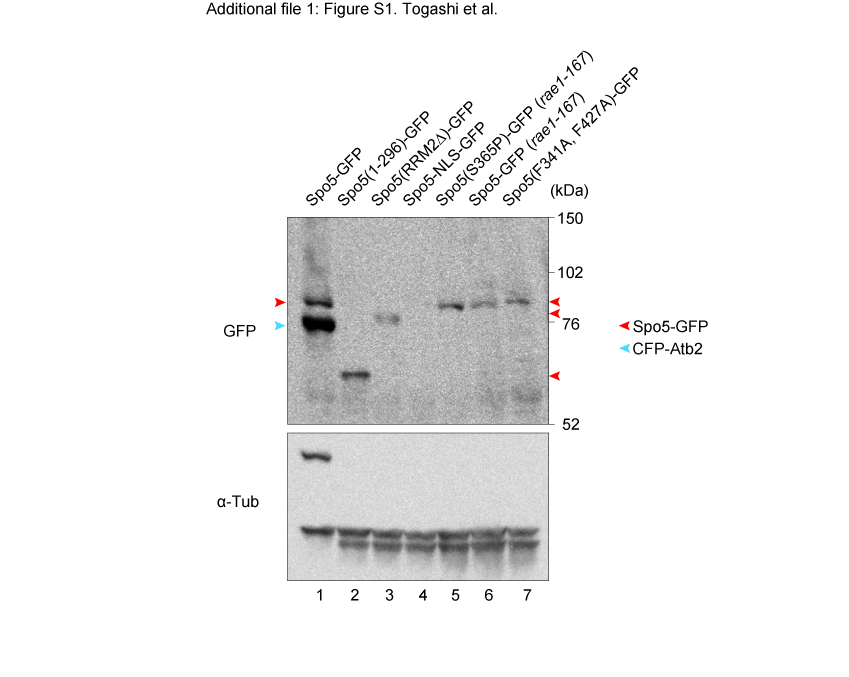

Supplement: Additional file 1: Figure S1 — Expression level of mutant Spo5 proteins under meiotic conditions. Cell extracts were prepared from representative spo5 mutant strains incubated on SPA at 25 ºC for 8 hours. They were separated by SDS-PAGE and subjected to western blotting. Each Spo5-GFP protein, which was expressed from the authentic spo5 promoter, was detected with an anti-GFP antibody. α-tubulin was also detected as a loading control. The wild-type strain shown here for comparison carried an additional gene encoding CFP-tagged Atb2, which reacted with both anti-GFP and anti-α-tubulin (lane 1). [file 1471-2180-14-188-S1.tiff]

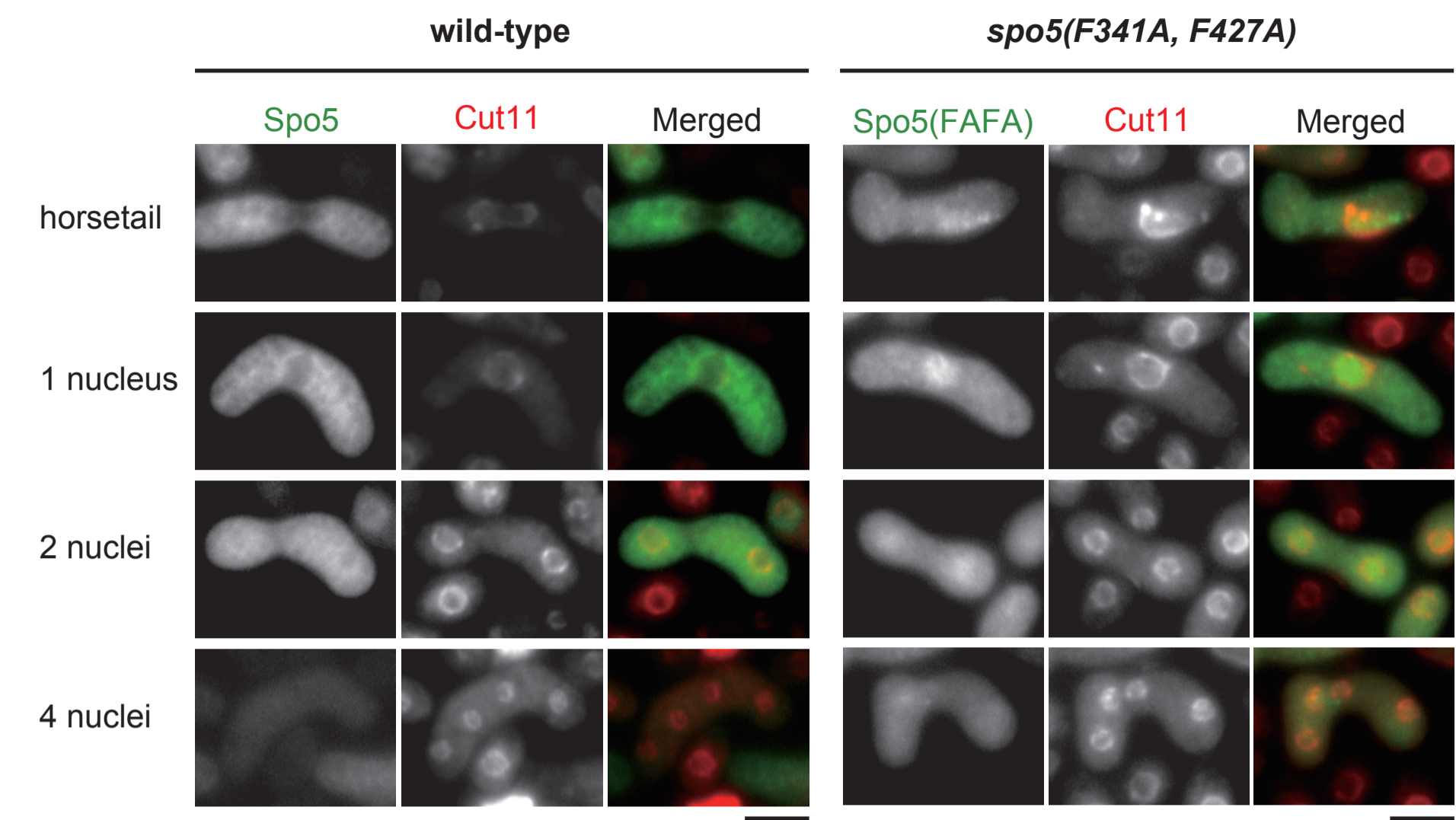

Supplement: Additional file 2: Figure S2 — Localization of Spo5(FAFA) protein during the progression of meiosis. Localization of WT Spo5 (left) and Spo5(FAFA) (right) was examined at four stages of meiosis, namely horsetail-movement, one-nucleus, two-nuclei, and four-nuclei stages. GFP-tagged Spo5 (green) and the nuclear envelope marker Cut11-4mRFP (red) were detected. Scale bar, 5 μm. [file 1471-2180-14-188-S2.pdf]

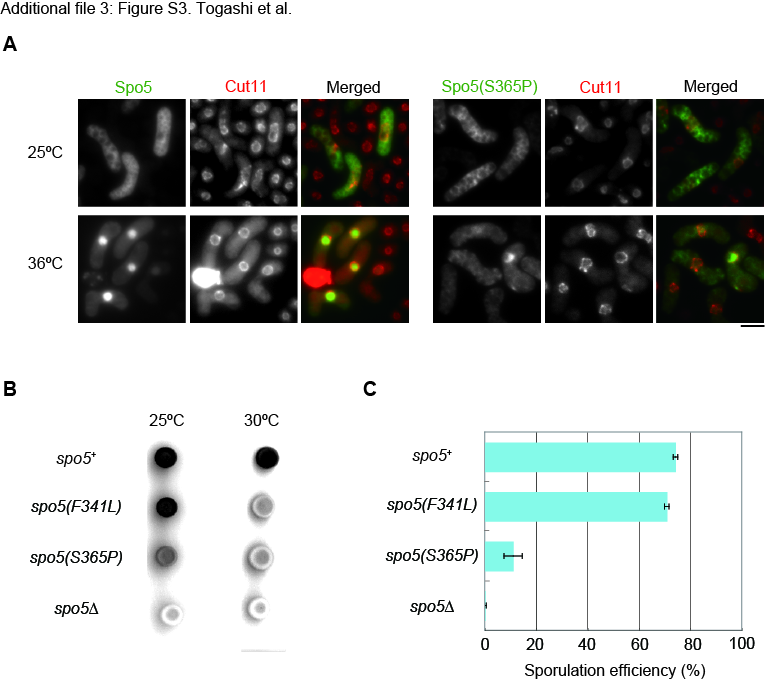

Supplement: Additional file 3: Figure S3 — Characterization of a novel spo5 missense mutant spo5(S365P). (A) Spo5(S365P)–GFP did not accumulate in the nucleus of the temperature-sensitive rae1-167 cells. Cells were incubated at 25°C for 6 h and then shifted to 36°C for 3 h. Cut11-4mRFP was used as a nuclear envelope marker. (B) At 25°C, WT and spo5(F341L) cells were stained dark brown with iodine vapor and sporulated efficiently, while spo5(S365P) cells were stained light brown and sporulated weakly. The spo5∆ cells showed a white colony and did not sporulate. At 30°C, the two mutants showed nearly white colonies and did not sporulate efficiently, as indicated quantitatively in Figure 1D. (C) Sporulation efficiency of WT and the two spo5 mutant strains at 25°C (n > 500). Error bars indicate standard deviation. [file 1471-2180-14-188-S3.tiff]

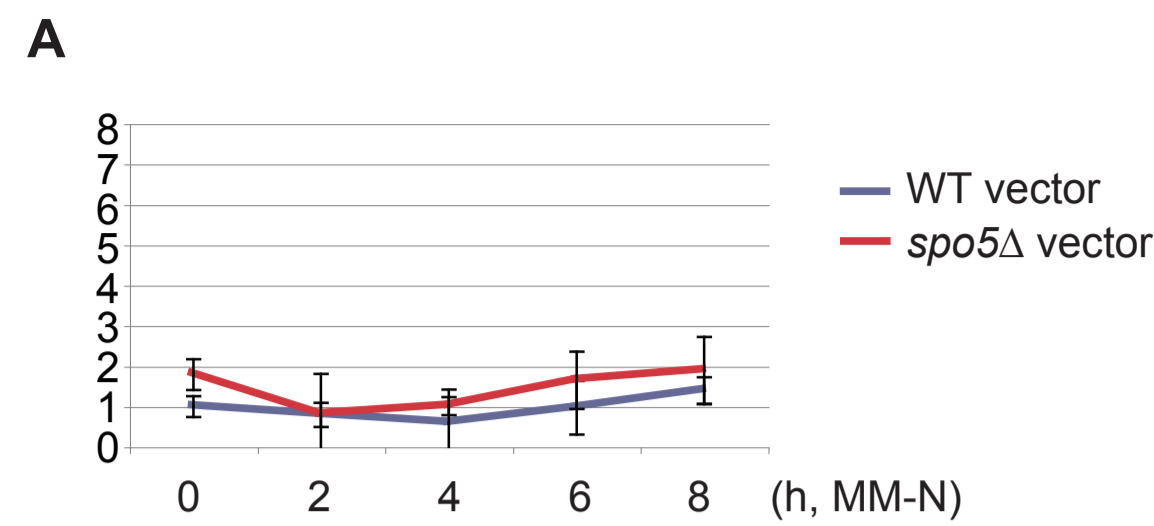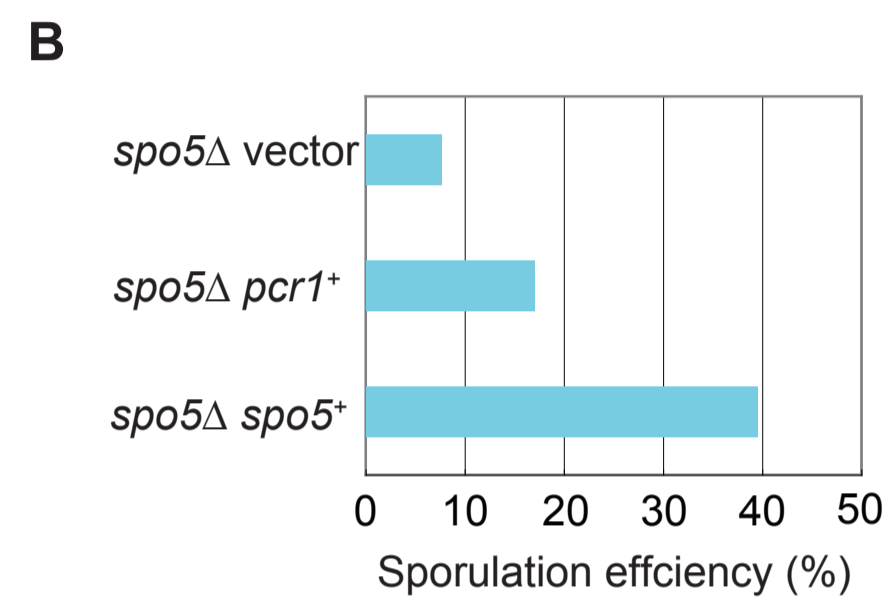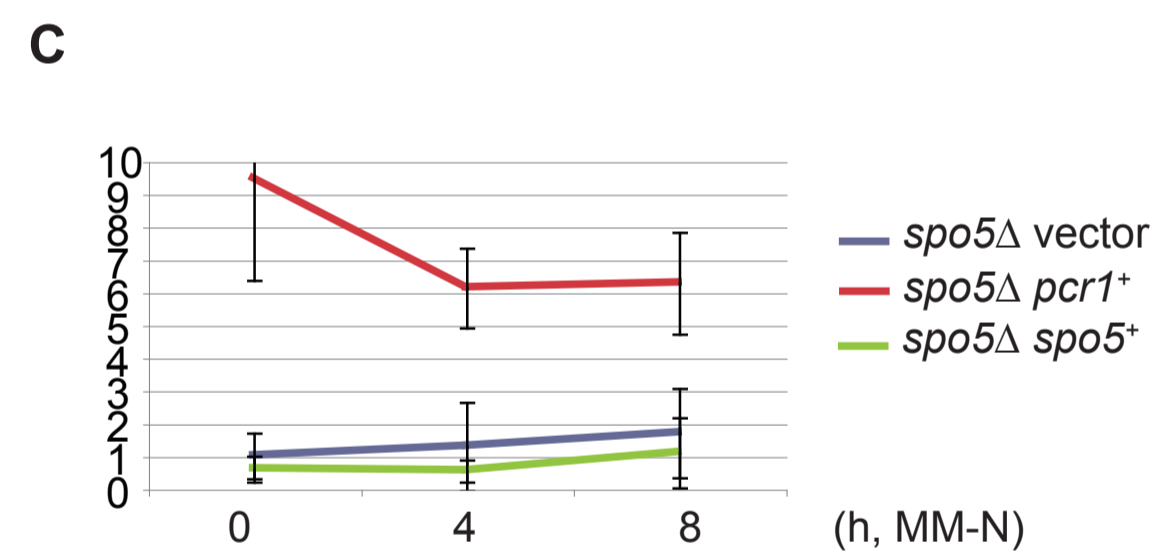

Supplement: Additional file 4: Figure S4 — Expression of pcr1+ mRNA is not lowered in spo5∆ cells. (A) Meiosis was induced in spo5+ (WT) and spo5∆ diploid cells, as in Figure 5B, and total RNA was isolated from them every 2 hours. The relative amount of pcr1+ mRNA in spo5+ and spo5∆ cells was determined by RT-qPCR. The quantity of mRNA was normalized to the expression level of act1 mRNA. Error bars show standard deviation. Two independent samples were analyzed for each strain. n = 3, for each sample. (B) Sporulation efficiency was measured for spo5∆ cells harboring either the vector, the pcr1 + clone, or the spo5 + clone. (n > 500) (C) The relative amount of pcr1+ mRNA was determined by RT-qPCR in spo5∆ cells harboring either the vector, the pcr1 + clone, or the spo5 + clone. Total RNA was isolated every 4 hours after the induction of meiosis, and analyzed as in (A). Error bars show standard deviation. [file 1471-2180-14-188-S4.pdf]
